# Whole genome sequencing as a reliable alternative for *Salmonella* serotyping: a comparative study with the gold-standard method

**DOI:** 10.3389/fmicb.2025.1685741

**Published:** 2025-11-26

**Authors:** Irene Mileto, Greta Romano, Stefano Gaiarsa, Giulia Grassia, Jessica Bagnarino, Antonio Piralla, Vincenzina Monzillo, Patrizia Cambieri, Fausto Baldanti, Marta Corbella

**Affiliations:** 1 Department of Microbiology and Virology, Fondazione IRCCS Policlinico San Matteo, Pavia, Italy; 2 School of Specialization in Microbiology and Virology, University of Pavia, Pavia, Italy; 3 Department of Clinical, Surgical, Diagnostic and Pediatric Sciences, University of Pavia, Pavia, Italy

**Keywords:** *Salmonella enterica*, whole genome sequencing (WGS), serotyping assay, SeqSero2, SISTR

## Abstract

**Introduction:**

*Salmonella* is a major foodborne pathogen of significant global health concern, leading to conditions like gastroenteritis, with symptoms such as diarrhea, abdominal cramps, and fever. Although most cases are self-limiting, *Salmonella enterica* can cause bacteremia or typhoid fever in some cases. There are over 2600 known serotypes of *S. enterica*. Each serotype is distinguished by differences in its surface antigens (somatic O and flagellar H), which is the basis for their classification. Considering that traditional phenotypic typing is laborious and time-consuming, the goal of this study is to evaluate whole genome sequencing for *S. enterica* classification.

**Methods:**

From January 2021 to December 2024, 282 isolates of *S. enterica* were collected at the Microbiology and Virology department of Fondazione IRCCS Policlinico San Matteo (Italy). The isolates were serotyped for O and H antigens with the gold-standard serum agglutination method. Whole genome sequencing. WGS was performed using Illumina MiSeq and quality-filtered reads were used to perform serotype prediction using SeqSero2 and SISTR tools.

**Results:**

The genomic typing of both tools shows an optimal concordance level with the gold-standard method, accurately predicting the serotype (both O and H antigens) for 236 out of 282 analyzed isolates. The discordance rate was 12 isolates out of 282. In addition, 34 isolates were correctly typed by the genomic methods, whereas typing with the gold-standard was unsuccessful. Genomic typing was able to classify all isolates while staying as close as possible to the present classification of *Salmonella* into subspecies and serovars.

**Discussions:**

Whole genome sequencing has proven to be a robust and reliable method for *Salmonella* typing. It allows for the simultaneous analysis of multiple samples and shortens processing times. Moreover, WGS enabled the recovery of identifications that could not be obtained using the gold-standard method.

## Introduction

*Salmonella enterica* is a major global cause of foodborne illness, with clinical manifestations influenced by bacterial load, host immune status, and serotype. Human infection is primarily associated with consumption of contaminated food (Carrera et al., 2023; European Centre for Disease Prevention and Control [ECDC], 2022; Huedo et al., 2017; Pagani et al., 2023; Petrin et al., 2024).

The genus *Salmonella* comprises two species, *S. bongori* and *S. enterica*, the latter of which is subdivided into six subspecies encompassing over 2,600 serovars, classified by somatic (O) and flagellar (H) antigens (Dos Santos et al., 2019). *S. enterica* subsp. *enterica* (subsp. I) includes approximately 1,600 serovars, of which only 32 demonstrate key features of pathogenicity, environmental persistence, and inter-ecosystem transmission, due to food handling and processing (Agbaje et al., 2011; Tack et al., 2020). Twelve serovars, including S. Enteritidis, S. Typhimurium, and S. Monophasic Typhimurium (*4,[5],12:i:-*), are implicated in ∼90% of foodborne outbreaks (European Food Safety Authority [EFSA] and European Centre for Disease Prevention and Control [ECDC], 2024).

Non-typhoidal *Salmonella* (NTS) is the second most reported zoonotic infection in the European Union (EU), accounting for the majority of foodborne outbreaks (European Food Safety Authority [EFSA] and European Centre for Disease Prevention and Control [ECDC], 2024). In 2023, 77,486 salmonellosis cases were reported, up from 65,478 in 2022. Effective prevention requires early detection across the food chain, from animal feed and livestock to processing and retail (Centers for Disease Control [CDC], 2013). Notification of NTS is mandatory in most EU countries, supported by surveillance systems such as Enter-Net Italy.

Serotyping is based on the Kauffmann-White-Le Minor (KW) scheme, which distinguishes antigenic profiles via slide agglutination using specific antisera targeting 64 O and 114 H antigen variants (Grimont and Weill, 2007). The sero-agglutination test is the standard method. The model relies solely on observable phenotypic traits, which is therefore reflected in a subjective interpretation, which requires correctly trained workers. However, the agglutination method has several limitations, including (i) weaker and non-specific agglutination reactions that can lead to false-positive results; (ii) rough, non-motile, and mucoid strains that exhibit autoagglutination and loss of antigen expression; (iii) the technique necessitates the use of more than 150 highly specific antisera (Schrader et al., 2008).

The introduction of Next Generation Sequencing (NGS) and the decrease of sequencing expenses have made whole genome sequencing (WGS) more affordable, and it now functions as a substantial tool for pathogen subtyping, source tracing, and characterization, including virulence and antimicrobial resistance gene profiling (Deng et al., 2015). The use and application of WGS, including Salmonella serotyping, in routine laboratories is becoming a viable option. WGS offers superior discriminatory power over the gold-standard agglutination method because it enables rapid, high-resolution pathogen characterization and enhances outbreak source tracing, representing a transformative tool in Salmonella epidemiology surveillance (Deng et al., 2015; Koser et al., 2012).

Several studies have evaluated the use of WGS for *Salmonella* serotyping, assessing its reliability, performance, and applicability as an alternative to traditional phenotypic methods (Cooper et al., 2020; Ibrahim and Morin, 2018; Jibril et al., 2020; Robertson et al., 2018; Uelze et al., 2020; Wu et al., 2021; Xu et al., 2020; Yachison et al., 2017). These works collectively highlight the potential of WGS to provide accurate and high-throughput serotype prediction, streamline laboratory workflows, and support large-scale epidemiological investigations. However, the analysis of genomic data and the accurate identification of isolates are strongly influenced by the choice of bioinformatic tools. Among those most commonly employed, SeqSero2 and SISTR are widely used (Yoshida et al., 2016; Zhang et al., 2019). The extensive use of WGS for the identification of *Salmonella* serovars has led to the accumulation of *Salmonella* genomes in comprehensive databases such as NCBI’s Pathogen Detection (comprising approximately 765,000 *S. enterica* genomes) and EnteroBase (with over 704,000 genomes), which are regularly updated to support ongoing surveillance and research (Dyer et al., 2024; The NCBI Pathogen Detection Project, 2016; Robertson et al., 2018).

The aim of this study was to assess the concordance between the gold-standard sero-agglutination method and two alternative approaches, WGS combined with SeqSero2 and SISTR, for the routine serotyping of *Salmonella* isolates in our hospital.

## Materials and methods

### Sample collection and Kauffmann-White-Le Minor serotyping

A total of 282 *Salmonella* isolates were collected from 2021 to 2024 at the Microbiology and Virology Unit of Fondazione IRCCS Policlinico San Matteo, a 900-bed hospital in Northern Italy.

Clinical samples (feces, blood, urine, synovial fluid and abscess) were cultured according to laboratory protocols and genus identification was confirmed by matrix-assisted laser desorption ionization time-of-flight (MALDI-TOF) mass spectrometry (Bruker Daltonik GmbH, Bremen, Germany) and Bruker BioTyper database version 3.1. The isolates were serotyped with rabbit antisera (SSI Diagnostica A/S, Denmark) following the traditional KW phenotypic method based on Ryan’s scheme (Ryan et al., 2017). The serotype was determined by agglutinating pure colonies with polyvalent and then monovalent antisera to determine the somatic antigen (O) and then to characterize the flagellar antigens (H). This provided the antigenic formula of the isolate associated with the serotype name and subspecies.

### Whole genome sequencing

The genomic DNA extraction was performed by using the QIAsymphony DSP Virus/Pathogen Midi Kit (QIAGEN, Germany), according to the manufacturer’s instructions. DNA was quantified by fluorescence, using the Qubit 4.0 fluorometer kit (Invitrogen, Carlsbad, CA, United States). All isolates underwent WGS on the Illumina MiSeq platform (San Diego, California, United States) performed with a 2 × 250 bp paired-end sequencing run. Libraries were prepared according to the manufacturer’s protocol using the Nextera XT DNA library preparation kit to ensure optimal fragment size distribution and sequencing quality. The evaluation of raw sequencing data integrity was performed using the FastQC tool (Andrews, 2010). Raw reads with Phred scores below 20 were removed using the fastp tool (Chen, 2023) to include only high-quality reads.

### Serovar prediction from sequencing data

The SeqSero2 and SISTR tools were used to determine the serotype of the *Salmonella* isolates (Yoshida et al., 2016; Zhang et al., 2019). Paired-end FASTQ reads were used as input for the allele micro-assembly workflow of SeqSero2 (version 1.2.1) (Zhang et al., 2019), which predicts the serovar based on the detection and combination of specific genetic determinants associated with the O (somatic) and H (flagellar) antigens. Consensus sequences of the *Salmonella* isolates were generated using the Shovill tool (Shovill, 2020; Supplementary Table 1). The resulting FASTA files were subsequently analyzed using the SISTR tool (Yoshida et al., 2016), which similarly infers the serotype through gene presence and sequence analysis of loci involved in antigen biosynthesis and expression. The results from the traditional *Salmonella* serotyping and from WGS were analyzed for their differences and similarities using an in-house R script to generate figures (libraries: tidyverse, stringr, data.table, ggplot2, ggnewscale, RColorBrewer, Biostrings, reshape2, plyr) (RStudio Team, 2015).

McNemar’s chi-squared test was used to assess the statistical significance of the classification performance between SeqSero2 and SISTR versus KW methods.

## Results

### The *Salmonella* specimen cohort

Among the 282 clinical samples analyzed, fecal specimens accounted for 80.5% (*n* = 227) of the total. Blood and urine samples represented 9.6% (*n* = 27) and 8.1% (*n* = 23), respectively. The remaining five samples included abscess material (*n* = 3), biopsy specimen, and synovial fluid (Supplementary Table 2). The latest ECDC report on salmonellosis reports an NTS invasiveness rate of around 6% (European Centre for Disease Prevention and Control [ECDC], 2022). However, the latest data collected by the Enter-Net Italia system (National Surveillance 2016–2021 of *Salmonella*, *Campylobacter*, *Shigella*, and *Yersinia* infections) shows that approximately 8.3% of isolated *Salmonella* strains cause invasive infections (Istituto Superiore di Sanità, 2024). This study highlights a higher rate of invasiveness among NTS strains compared to both European and Italian data.

**FIGURE 1:**
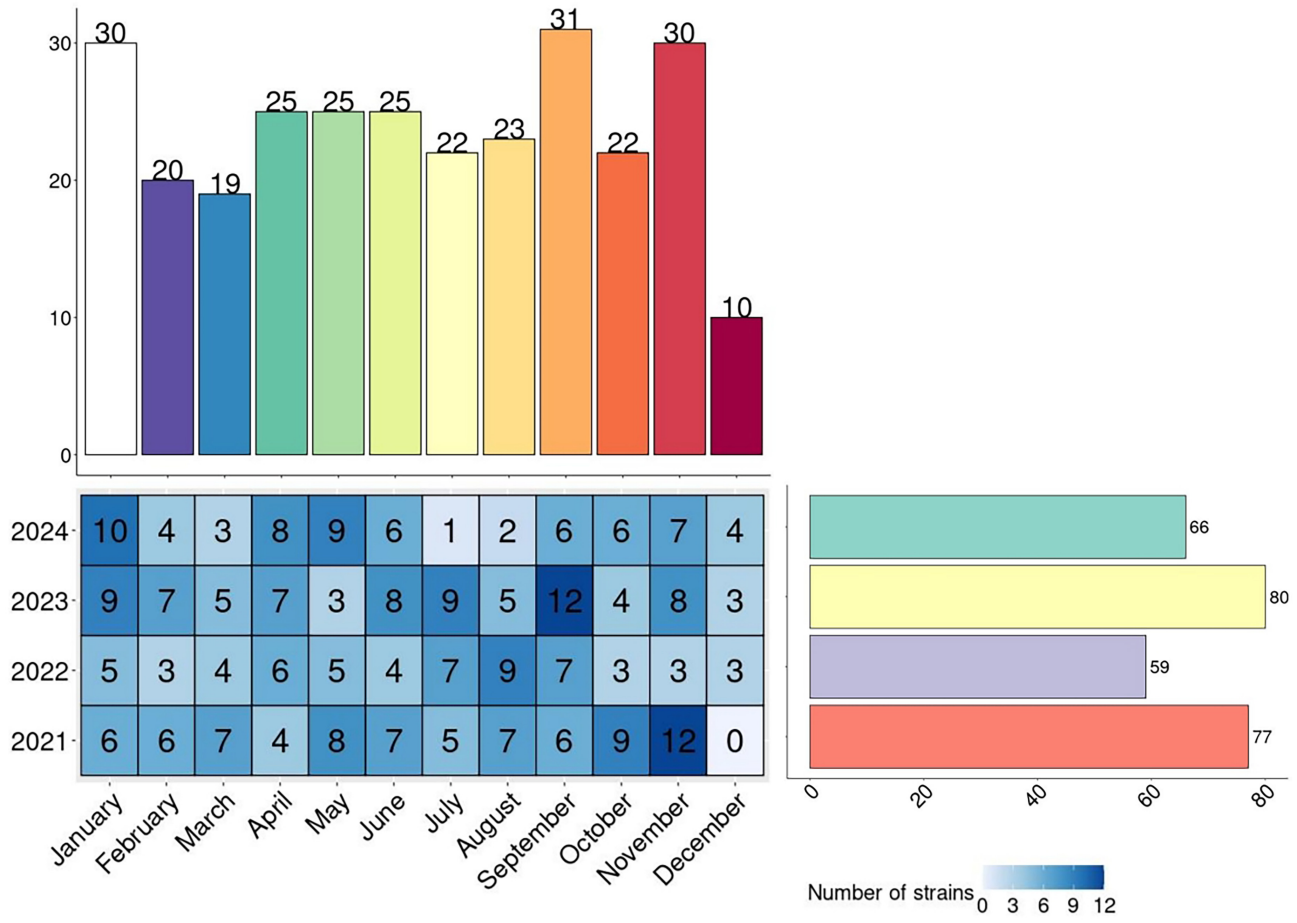
Distribution over the period 2021–2024 of samples positive for *Salmonella spp*. The heatmap shows the number of *Salmonella* isolates for each month and year. Barplots are cumulative for year and month.

The average annual number of samples was 70 per year. No monthly peak was observed in the sample collection, as it remained constant throughout the years (Figure 1).

**FIGURE 2:**
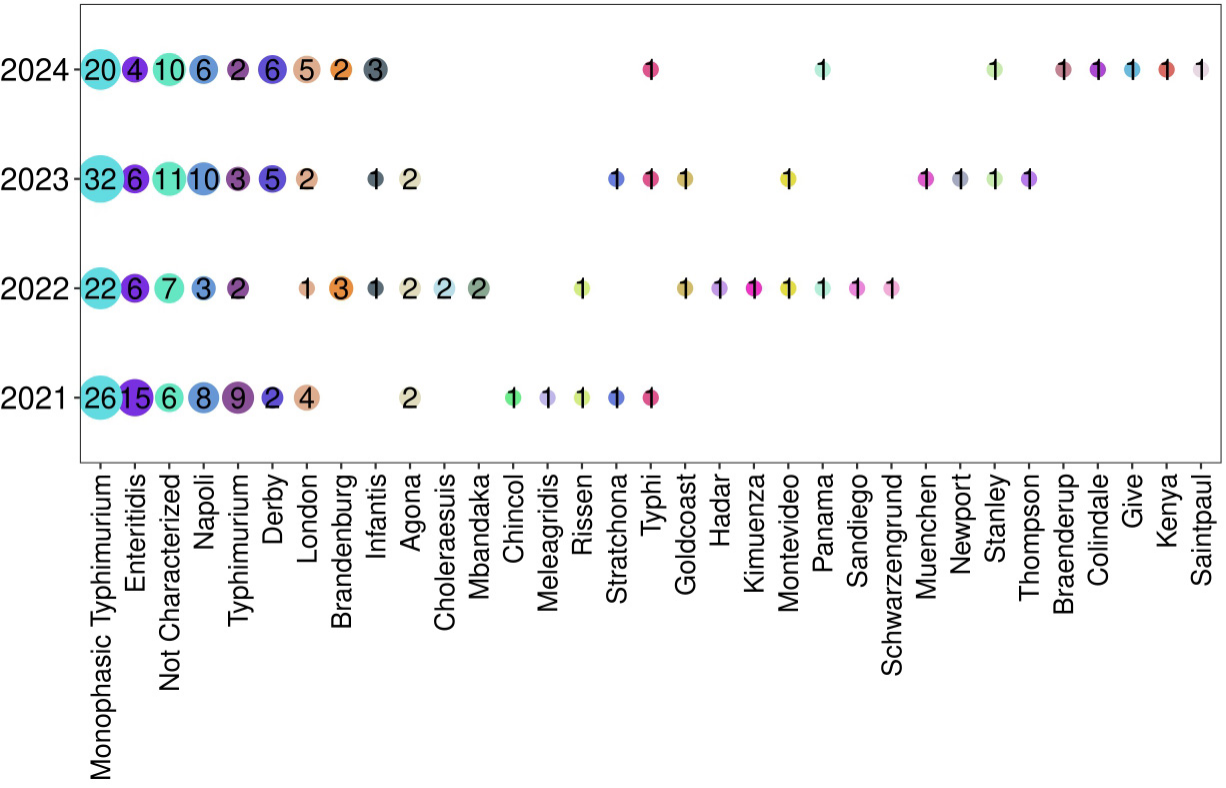
Prevalence of *Salmonella spp* in the period 2021–2024. The frequency of all serovars is reported in Supplementary Table 3.

The sero-agglutination of 282 isolates showed 32 different *Salmonella* serovars, among which S. Monophasic Typhimurium was the most prevalent (Figure 2 and Supplementary Table 3). This serovar was identified in 100 of the 282 isolates (35.4%) followed by serovar *Enteritidis* in 31 (10.9%) isolates. All other serovars were detected at frequencies below 10%. In addition, the monthly distribution of the two most prevalent serovars was analyzed for the years 2021–2024, but no distinct monthly peaks in prevalence were observed (Supplementary Figure 1).

Finally, serotyping was not assessed in 12% of the isolates (*n* = 34) because of the presence of autoagglutination or non-agglutination isolates or the non-availability of certain specific antisera in our laboratory (Wattiau et al., 2011).

For serovars causing sepsis, no distinct trend was observed, and the overall numerical pattern recorded at our hospital was confirmed. Among the total isolates, six isolates of *S*. Monophasic Typhimurium, four isolates of *S*. Enteritidis, and two isolates of *S*. Napoli were responsible for invasive infections (Supplementary Table 2). Additionally, two cases of typhoid fever were reported, with *S*. Typhi isolated exclusively from blood cultures.

### Comparison WGS and gold-standard method

**FIGURE 3:**
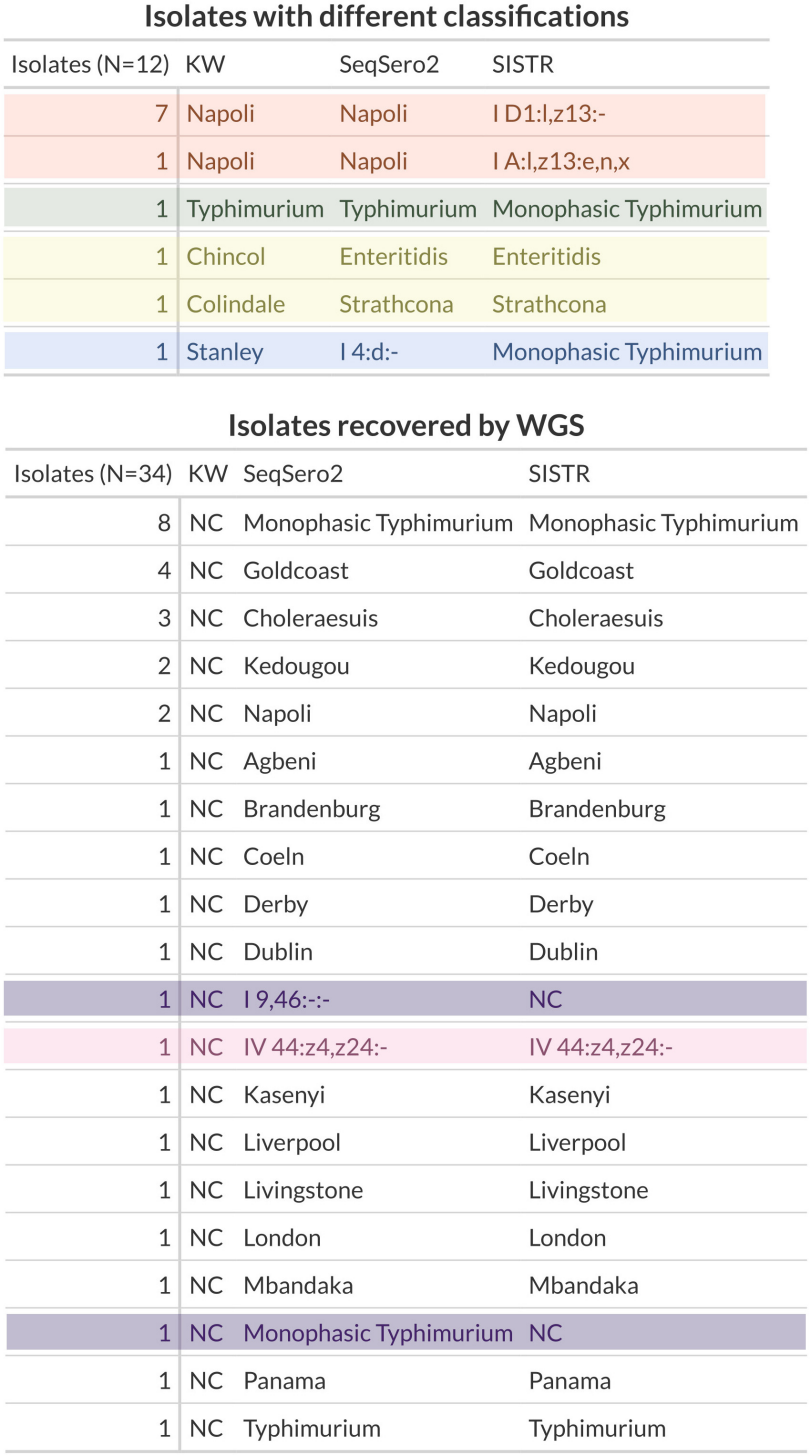
Discrepant classifications for the three methods (KW, SeqSero2, and SISTR) across *Salmonella enterica* isolates. Upper table: isolates with different classification among the three methods. Bottom table: isolates recovered by WGS and not classified by KW. NC, not characterized.

Of the 282 samples analyzed, both the gold-standard method and WGS successfully assigned the isolates to a specific serovar or antigenic profile in 248 cases (88%), whereas for the remaining 34 isolates, only WGS was able to achieve the identification (Figure 3, bottom table).

Comparing the predicted *Salmonella* serotypes derived from the sequencing protocol with respect to the gold-standard, both SeqSero2, SISTR and KW serotyping predicted identical *Salmonella* serotypes in 236 out of 248 isolates, showing 95% of concordance.

In the remaining 12 cases (5%), both methods (KW and SeqSero2/SISTR) achieved classification and yielded discordant results (Figure 3, upper table).

Eight isolates were classified successfully by KW and SeqSero2 while SISTR was able to recover the antigenic profiles without the serovar identifier (Figure 3, upper table, orange box).

One sample was classified as Typhimurium by KW and SeqSero2 while SISTR identified it as Monophasic Typhimurium (Figure 3, upper table, green box).

Two isolates were phenotypically identified as *Salmonella* Chincol and Colindale, whereas the Enteritidis and Strathcona classifications were produced by WGS (Figure 3, upper table, yellow box).

The last isolate was classified by KW as Stanley, Monophasic Typhimurium by SISTR, whereas SeqSero2 could only identify the antigen (I 4:d:-) and not the classification (Figure 3, upper table, blue box).

Regarding the classification recovery rate, WGS assigned a specific classification to 34/282 (12%) isolates that were not characterized by KW (Figure 3, bottom table). In one of these cases, SeqSero2 and SISTR were able to recover only the antigenic profile without reaching classification (Figure 3, bottom table, pink box). Moreover, the SeqSero2 tool recovered a classification and an antigenic profile in the case of two isolates not classified by KW and SISTR, revealing high sensibility (Figure 3, bottom table, purple box).

McNemar’s chi-squared test revealed a statistically significant difference in classification performance between the KW and WGS-SeqSero2 method, with the latter outperforming the gold-standard approach (χ^2^ = 34, df = 1, *p* = 5.511 × 10^–9^).

McNemar’s chi-squared test between KW and WGS-SISTR method was also significant (χ^2^ = 32, df = 1, *p*-value = 1.542 × 10^–8^). Overall, WGS significantly reduced the proportion of untyped isolates compared with KW.

Regarding the comparison between SeqSero2 and SISTR, the test resulted in a non-significant *p*-value (χ^2^ = 2, df = 1, *p* = 0.1573), indicating that there are no statistically significant differences between the two methods in the sample identification rate.

## Discussion

In Italy, non-typhoidal *Salmonella* is the second most prevalent infection linked to diarrhea. *S*. Monophasic Typhimurium was the most prevalent serotype associated with non-typhoidal salmonellosis in Italy, which is in contrast with the European trend reported by Centers for Disease Control [CDC] (2013). *S.* Enteritidis is the most prevalent serotype in Europe, while in our hospital, as well as in the rest of Italy, ranks second.

Determining the phenotype of the isolates is a labor-intensive process that requires a significant amount of time, typically taking up to three days of active work of a trained operator to complete, and this applies to only a limited number of isolates to be typed. The turnaround time for WGS can vary depending on the library preparation kit and the sequencing platform, as well as the possibility to automate the library preparation process. A single MiSeq run (500 cycles paired-end) comprising 16 bacterial genomes generally takes 48–72 h (McGann et al., 2016) for Nextera XT protocol library by-hand preparation, sequencing, and data analysis, with 50% of active hands-on work time. Importantly, sequencing allows for a high-throughput, parallel processing of multiple genomes simultaneously, enabling the analysis of many isolates at once and obtaining much more information than sample typing (e.g., species, subspecies, serovar, virulence, pathogenicity and antimicrobial resistance), a scale that is not achievable with conventional phenotypic method (Ibrahim and Morin, 2018).

Certain *Salmonella* isolates require repeated subculturing in semi-solid media to enhance motility and flagellar antigen expression (Centers for Disease Control [CDC], 2013). Specific genetic mutations, such as single nucleotide polymorphisms can lead to the loss of serotype antigen expression, further limiting the reliability of traditional serotyping methods (Li et al., 2017). Conventional serotyping is also resource-intensive, necessitating the maintenance of a broad and diverse panel of antisera.

Recently, WGS has been applied in the area of *Salmonella* subtyping and has the potential to be a more dependable and efficient method (Ashton et al., 2016; Basso et al., 2025; Brümmer et al., 2024; Han et al., 2024; Morton et al., 2024). Tools like SeqSero2 and SISTR can forecast most *Salmonella* serotypes using large-scale genome sequencing data from databases containing *Salmonella* serotype determinants.

We performed the WGS approach and KW phenotypic method for 282 *Salmonella* isolates. The raw WGS data was analyzed using the two tools that have shown the best performance in literature, SeqSero2 and SISTR (Uelze et al., 2020). For 248 *Salmonella* isolates, both KW and WGS were able to achieve a classification or a profile antigen identification. WGS successfully predicted 236/248 (95%) isolates that shared the same antigenic structure and serotype classification of KW method. Overall, the discordance rate between the gold-standard method and WGS was 5% (12 isolates, Figure 3, upper table).

The discrepancies observed were mostly attributable to inherent differences between genotypic and phenotypic approaches, with WGS-based tools relying on the detection of antigen-encoding genes and KW depending on antigen expression and agglutination patterns. In eight samples, SISTR failed to return a serovar classification describing only an antigen profile (Figure 3, upper table, orange box). Rather than being associated with lower sensitivity of SISTR, the discrepancy may arise from both methodological differences among the tools. SISTR integrates information from antigen gene prediction with core genome multilocus sequence typing (cgMLST) to infer the most likely serovar. SeqSero2 relies solely on the presence and combination of antigen-encoding genes (O- and H-antigen loci), while KW classification is based on phenotypic expression of these antigens.

In the second case (Figure 3, upper table, green box), KW and SeqSero2 may have detected partial sequences or traces of the second phase, classifying the isolate as biphasic. Instead, SISTR may be more stringent and classify it as monophasic if the phase 2 gene is too divergent. However, since SISTR classification relies on a consensus derived from other tools, the divergence could be due to a different assembly.

The most likely reason for the difference in KW phenotypic identification with respect to WGS classification in the third and fourth cases (Figure 3, upper table, yellow box) is that the two serovars share highly similar or identical antigenic factors for one phase but differ in antigens. A weakly expressed or absent flagellar/somatic antigen could result in phenotypic agglutination matching Colindale’s known antigen formula, even if it carries Strathcona-specific genes. Moreover, the antisera used in the KW scheme can sometimes cross-react with antigens from closely related serovars, leading to misidentification if the full antigenic profile is not clearly expressed (Figure 3, upper table, yellow box).

In the last case, WGS-based algorithms may have assigned a different or more generic genetic profile depending on sequence homology and database composition. Thus, if antigen expression is altered, the phenotype may not correspond to the genotype, and the observed antigenic profile could be more readily matched to Stanley in traditional reference charts (Figure 3, upper table, blue box).

Overall, the disparity between genotypic and phenotypic expression and the principle underlying the two methods can be decisive in identifying and classifying the antigenic structure.

WGS enabled the characterization of 34 isolates out of 282 (12%) that were untypeable by conventional methods (Figure 3, lower table). However, it should be noted that of the two tools, SeqSero2 performed slightly better, recovering one classification and one antigenic profile (34/34, 100%) compared to SISTR (32/34, 94%) (Figure 3, lower table, purple boxes). This suggests that SeqSero2 may be more sensitive in detecting partial or divergent antigen gene sequences, while SISTR’s more conservative assignments could be advantageous in contexts where minimizing potential misclassification is a priority. However, the overall performances of SeqSero2 and SISTR were similar to each other as previously demonstrated from different studies (Ashton et al., 2016; Uelze et al., 2020; Wu et al., 2021; Zhang et al., 2015, 2019). Indeed, in the comparison of several open-source tools for serotyping *Salmonella* spp., SISTR and SeqSero2 produced the most accurate and reliable results, predicting the serovars of 94% and 87% of all isolates, respectively, as reported by Uelze et al. (2020).

The statistically significant differences observed in McNemar’s chi-squared tests support the superior performance of WGS over the KW method in *Salmonella* classification for both tools (SeqSero2: *p* = 5.511 × 10^9^, SISTR: *p* = 1.542 × 10^8^). Moreover, the comparison between SeqSero2 and SISTR yielded a non-significant *p*-value (*p* > 0.05), indicating that there are no statistically significant differences between the two methods in sample identification rates. Our laboratory consistently employs WGS to predict *Salmonella* serotypes for each isolate from ongoing investigations, as well as from previously archived isolates dating back to previous years.

Despite the demonstrated accuracy and efficiency of the WGS tool for *Salmonella* serotyping, certain serovars remain absent from the SeqSero2 and SISTR databases, leading to occasional discrepancies between genomic and traditional serotyping results. It is anticipated that as these databases are progressively expanded, it will be possible to overcome these limitations. Overall, this research confirmed that genomic serotyping is a valuable support for traditional phenotypic methods.

### Study limitations

A limitation of this study is the lack of demographic data that limited our ability to explore possible associations between serovars and specific patient characteristics, infection sources, or clinical outcomes. Moreover, the small number of non-intestinal isolates in our dataset may have reduced the representativeness of invasive or systemic infections. The incomplete panel of antisera also introduced potential biases in the comparison between phenotypic and genomic serotyping methods, as some discrepancies could not be fully resolved through traditional serological testing. Future studies integrating comprehensive epidemiological metadata, a larger number of isolates from diverse clinical and environmental sources, and complete serological characterization will allow a more accurate assessment of the relationships between genotype, phenotype, and epidemiological patterns.

## Conclusion

In conclusion, this study underscores the substantial advantages of WGS for the serotyping of *Salmonella* isolates, presenting a robust and efficient alternative to conventional phenotypic methods. Phenotypic methods for *Salmonella* identification exhibit inherent limitations due to their reliance on manual operator interpretation, which introduces subjectivity and potential for error. The requirement for skilled personnel to perform and interpret serological assays contributes to variability in results and can reduce reproducibility across laboratories. Additionally, these methods are labor-intensive and time-consuming, further delaying timely results. Phenotypic approaches often lack the resolution to discriminate closely related isolates, impeding accurate outbreak investigations and epidemiological surveillance. These challenges underscore the necessity for integrating high-resolution, automated molecular techniques such as WGS to enhance accuracy, standardization, and timeliness in *Salmonella* surveillance (Dyer et al., 2024). The 12% recovery rate of untyped isolates highlight the method’s potential to refine and streamline laboratory workflows emerging as a reliable and scalable approach for *Salmonella* surveillance. Its integration into routine laboratory operations offers notable benefits, including reduced labor demands, enhanced quality control, and expedited reporting. This work also establishes a solid basis for implementing *Salmonella* surveillance in our hospital and enables more timely reporting of identified serovars to the EnterNet-Italy platform (Istituto Superiore di Sanità, 2024), which coordinates national surveillance efforts. Finally, this approach contributes to national and global strategies for the tracking, prevention, and control of salmonellosis.

## Data Availability

All FASTQ files used in these analyses have been deposited in the GenBank Sequence Read Archive (SRA) (PRJNA1307988).

## Supplementary material

Supplementary Figure 1 Monthly distribution per year of the two most prevalent serovars, *S.* Monophasic Typhimurium and Enteritidis.

Supplementary Table 1 Assembly metrics of sequenced isolates.

Supplementary Table 2 Distribution of serovars per specimen.

Supplementary Table 3 Frequency of serovars per year.
