# Supplementary material for: Whole genome sequencing as a reliable alternative for Salmonella serotyping: a comparative study with the gold-standard method
Source: Front Microbiol. 2025 Nov 26;16:1685741. doi: 10.3389/fmicb.2025.1685741 (PMC12689982; doi:10.3389/fmicb.2025.1685741)
Supplement: Supplementary Figure 1 — Monthly distribution per year of the two most prevalent serovars, S. Monophasic Typhimurium and Enteritidis. [file Data_Sheet_1.pdf]

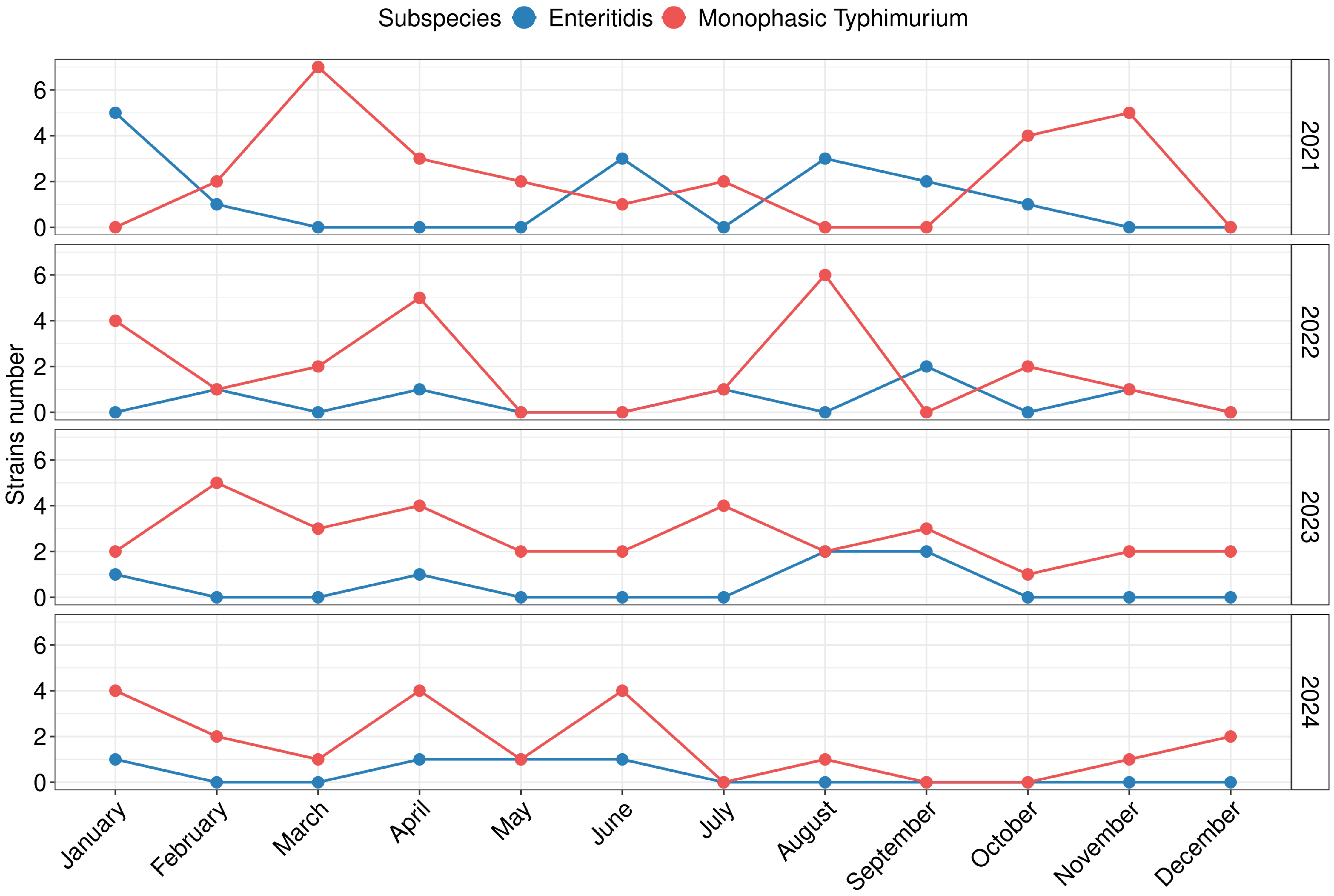

Supplementary Figure 1

Supplementary Table 1: Assembly metrics of sequenced isolates

	Genome size (bp)	Depth (X)	N50	N#Reads (PE)
1237S	4,744,589	62	531,842	1,596,898
1238S	4,744,539	56	401,090	1,475,034
1239S	4,745,002	55	327,422	1,409,958
1240S	4,744,376	62	401,090	1,658,150
1241S	4,668,964	49	432,535	1,470,450
1242S	4,744,888	67	401,380	1,655,226
1244S	5,057,467	68	189,151	1,884,800
1245S	4,796,964	76	401,380	3,199,896
1246S	5,048,357	74	277,439	1,980,132
1247S	4,952,955	89	279,039	2,419,856
1248S	5,055,760	71	184,487	1,948,250
1249S	4,971,178	58	301,490	1,863,172
1250S	4,901,227	48	223,309	1,269,066
1251S	4,952,875	75	319,550	2,535,966
1252S	4,971,493	81	270,584	2,148,306
1253S	3,408,413	5	4,787	146,794
1254S	5,043,399	63	270,584	1,803,232
1255S	5,006,536	73	270,466	1,948,912
1256S	4,920,588	80	270,591	2,634,602
1257S	5,124,569	67	217,076	1,834,006
1258S	4,725,319	36	140,519	1,200,168
1259S	4,925,842	49	282,781	1,385,938
1260S	2,682,967	5	3,975	154,522
1261S	5,151,541	76	239,264	2,302,416
1262S	4,976,347	68	204,596	1,878,288
1263S	4,792,172	50	301,490	1,350,454
1264S	4,958,029	30	316,485	981,988

Supplementary Table 1: Assembly metrics of sequenced isolates

	Genome size (bp)	Depth (X)	N50	N#Reads (PE)
1265S	4,969,821	76	280,701	2,060,158
1266S	4,701,380	49	441,790	1,436,950
1267S	4,951,604	87	225,887	2,548,564
1268S	4,608,826	72	432,808	2,008,190
1269S	4,598,066	60	335,472	1,487,406
1270S	4,709,172	53	490,280	1,418,818
1271S	4,700,370	48	460,245	1,284,556
1272S	4,701,443	72	478,906	1,919,056
1273S	5,135,225	65	600,120	1,920,474
1274S	4,542,397	57	151,374	1,483,652
1275S	4,953,714	82	282,781	2,145,350
1276S	4,533,583	80	141,264	3,251,424
1277S	4,708,784	44	409,740	1,150,956
1278S	5,043,555	81	270,584	2,423,368
1279S	5,043,234	54	270,584	1,519,496
1280S	4,531,300	48	141,265	1,190,130
1281S	4,707,678	80	401,085	2,497,788
1282S	4,687,054	62	135,222	1,864,172
1283S	4,813,374	80	252,059	2,216,512
1284S	4,707,117	64	479,296	1,797,138
1285S	4,703,151	80	479,332	3,132,986
1286S	4,546,409	82	151,420	2,548,082
1287S	5,167,707	62	423,839	1,674,670
1288S	4,899,339	75	185,363	2,226,266
1289S	4,708,486	82	490,293	2,593,204
1290S	4,554,929	78	118,296	2,263,188
1291S	4,754,144	62	96,799	1,707,246

Supplementary Table 1: Assembly metrics of sequenced isolates

	Genome size (bp)	Depth (X)	N50	N#Reads (PE)
1292S	4,771,599	56	659,115	1,453,716
1293S	4,740,574	84	479,333	2,349,422
1294S	4,726,911	57	368,131	1,429,902
1295S	4,654,233	77	151,241	1,931,276
1296S	5,108,517	68	227,551	2,180,710
1297S	4,703,026	64	490,721	1,876,236
1298S	4,876,399	85	270,591	5,045,386
1299S	4,692,844	60	505,837	1,562,832
1300S	4,983,899	68	282,781	1,954,458
1301S	4,948,985	74	277,466	2,110,440
1302S	5,035,456	71	217,076	2,002,092
1303S	4,741,374	87	543,233	2,845,434
1304S	4,926,038	75	276,391	2,091,296
1305S	4,918,735	69	261,757	2,287,258
1306S	4,721,570	72	355,392	1,926,960
1307S	4,755,978	79	398,795	2,289,910
1308S	5,036,733	76	217,076	2,308,894
1309S	5,035,006	54	224,122	1,513,138
1310S	4,749,284	70	307,519	1,921,570
1311S	5,082,507	75	270,473	2,090,564
1312S	5,034,886	59	217,076	1,731,688
1313S	5,188,782	89	349,321	2,453,212
1314S	5,187,348	78	358,397	4,151,264
1316S	4,838,483	75	180,522	1,954,842
1317S	5,054,457	85	270,588	2,290,844
1318S	3,762,463	5	5,300	136,928
1319S	4,965,609	70	270,591	1,878,552

Supplementary Table 1: Assembly metrics of sequenced isolates

	Genome size (bp)	Depth (X)	N50	N#Reads (PE)
1320S	5,036,526	59	165,326	1,819,768
1321S	5,071,542	60	217,076	1,656,868
1322S	4,710,886	76	479,296	1,892,234
1323S	3,849,747	5	5,306	142,110
1324S	5,058,233	62	400,308	1,803,648
1325S	4,953,153	61	239,074	1,684,346
1326S	4,954,618	73	270,591	2,019,638
1327S	3,959,298	5	5,762	163,022
1328S	4,719,342	92	276,820	2,350,156
1329S	4,993,835	79	270,473	3,540,744
1330S	4,953,308	65	251,346	1,679,154
1331S	4,996,493	91	238,416	2,454,162
1333S	5,035,386	75	217,076	2,131,176
1334S	4,928,650	78	277,132	3,195,832
1335S	3,663,184	5	4,735	159,506
1336S	4,802,736	9	12,954	289,586
1337S	2,634,437	5	4,163	155,222
1338S	4,589,808	66	596,258	1,822,438
1339S	4,833,977	78	83,990	2,745,114
1340S	4,707,693	63	197,737	1,727,134
1341S	3,011,734	5	4,241	149,620
1342S	4,911,989	70	251,421	2,113,800
1343S	3,988,490	6	6,588	169,300
1344S	4,739,286	83	490,287	2,238,980
1345S	5,028,484	75	256,009	2,209,356
1346S	5,028,771	57	256,009	1,727,838
1347S	2,744,015	5	4,127	133,038

Supplementary Table 1: Assembly metrics of sequenced isolates

	Genome size (bp)	Depth (X)	N50	N#Reads (PE)
1348S	4,752,064	83	371,828	2,269,184
1349S	4,595,624	71	551,252	1,734,134
1350S	4,927,549	79	223,309	2,051,780
1351S	4,954,883	75	165,416	2,340,436
1352S	4,885,629	81	277,465	2,548,310
1353S	1,693,177	4	2,725	102,800
1354S	4,967,538	72	276,215	2,873,634
1355S	5,036,564	55	277,465	1,680,954
1356S	4,887,052	67	277,465	2,641,784
1357S	4,976,691	85	277,466	2,491,784
1358S	4,443,083	7	7,694	188,136
1359S	4,880,538	75	261,757	2,140,156
1360S	4,700,015	39	461,666	1,165,842
1361S	4,711,046	85	492,194	2,408,010
1362S	4,805,859	65	366,021	1,716,304
1363S	4,749,397	79	439,227	3,250,966
1364S	4,612,919	74	110,004	1,743,790
1365S	5,050,335	85	273,060	4,015,854
1366S	4,579,412	74	145,067	1,811,178
1367S	4,983,814	72	170,436	2,025,324
1368S	5,084,271	47	226,358	1,406,170
1369S	4,621,520	81	366,933	2,208,422
1370S	4,893,209	67	251,387	2,085,336
1371S	4,621,138	85	400,853	2,116,680
1372S	4,781,521	91	169,004	2,374,912
1373S	4,731,739	57	510,287	1,726,634
1374S	4,879,424	41	191,318	1,280,096

Supplementary Table 1: Assembly metrics of sequenced isolates

	Genome size (bp)	Depth (X)	N50	N#Reads (PE)
1375S	4,785,693	64	170,545	2,161,894
1376S	4,940,941	68	148,598	2,119,960
1377S	5,205,569	79	358,367	2,903,418
1378S	4,942,094	47	278,118	1,314,574
1379S	4,622,407	84	252,023	2,202,938
1380S	4,951,084	69	193,423	2,022,412
1381S	4,718,227	62	340,270	1,923,520
1382S	4,701,826	56	479,332	1,480,924
1383S	4,622,238	82	341,294	2,339,658
1384S	4,621,712	82	341,294	2,300,374
1385S	4,961,960	63	281,520	1,801,964
1386S	5,012,028	78	210,691	2,390,548
1387S	5,017,254	53	282,781	1,534,586
1388S	4,884,875	51	149,102	1,623,134
1389S	4,943,094	62	270,473	2,049,148
1390S	4,998,747	54	123,707	1,691,388
1391S	5,021,940	85	277,466	2,404,540
1392S	4,882,170	53	130,055	1,652,996
1393S	4,929,018	53	97,113	1,642,336
1394S	5,015,791	52	251,286	1,386,594
1395S	4,952,556	68	239,267	2,148,604
1396S	5,037,173	72	210,373	2,094,056
1397S	4,985,930	66	196,363	1,779,420
1398S	5,071,588	58	270,591	1,676,844
1399S	4,891,929	88	170,845	2,374,346
1400S	4,916,692	64	167,913	1,882,946
1401S	4,993,843	82	165,051	2,351,680

Supplementary Table 1: Assembly metrics of sequenced isolates

	Genome size (bp)	Depth (X)	N50	N#Reads (PE)
1402S	4,950,727	71	282,781	1,969,766
1403S	4,707,815	74	439,135	2,248,134
1404S	5,149,804	73	270,466	2,693,950
1405S	4,919,608	92	276,391	2,630,588
1406S	4,599,526	83	129,980	2,137,562
1407S	4,856,636	85	121,749	3,536,054
1408S	4,918,730	66	188,041	1,915,896
1409S	4,935,580	51	282,781	1,529,672
1410S	4,877,489	71	432,618	2,012,332
1411S	4,637,849	67	237,541	1,914,254
1412S	4,757,292	61	101,437	1,667,064
1413S	4,515,794	71	88,955	1,796,400
1414S	4,758,163	81	101,436	2,841,176
1415S	4,771,081	38	231,594	1,113,014
1416S	4,507,590	56	61,797	1,811,308
1417S	4,699,579	44	460,572	1,316,378
1418S	4,836,070	45	398,728	1,290,884
1419S	5,126,072	80	282,781	2,401,348
1420S	4,937,293	48	316,045	1,440,476
1421S	4,911,977	52	259,544	1,371,884
1422S	5,115,612	60	282,782	2,054,256
1423S	4,708,839	61	409,929	1,993,220
1424S	5,029,157	82	177,737	2,687,010
1425S	4,825,338	73	431,130	2,246,838
1426S	4,734,242	86	401,198	2,242,234
1427S	4,733,787	81	439,123	2,106,218
1428S	4,928,520	49	317,065	1,501,918

Supplementary Table 1: Assembly metrics of sequenced isolates

	Genome size (bp)	Depth (X)	N50	N#Reads (PE)
1429S	4,732,115	72	262,260	1,792,786
1430S	5,294,972	71	164,138	2,224,640
1431S	4,700,556	49	265,909	1,335,258
1432S	4,976,499	71	263,408	1,904,106
1433S	4,528,857	41	151,422	1,111,222
1434S	4,999,985	47	224,122	1,484,896
1435S	4,638,397	37	151,420	1,190,684
1436S	4,767,747	48	157,293	1,481,668
1437S	4,597,769	37	408,114	1,124,286
1438S	5,027,401	59	217,077	1,577,300
1439S	4,530,941	40	145,340	1,224,832
1440S	4,597,649	45	439,291	1,316,974
1441S	4,690,567	47	464,709	1,390,486
1442S	4,523,261	71	141,242	2,056,954
1443S	4,704,095	55	151,455	1,708,224
1444S	4,749,318	67	657,597	2,269,240
1445S	4,972,058	59	316,045	2,049,330
1446S	4,742,600	69	543,182	2,327,824
1447S	4,528,854	55	151,423	1,745,272
1448S	4,723,046	76	355,392	2,670,860
1449S	4,925,384	91	270,584	2,389,838
1450S	4,861,216	53	417,760	1,801,544
1451S	4,923,992	47	270,584	1,518,382
1452S	4,975,173	61	440,849	2,101,898
1453S	4,922,072	67	277,466	2,034,324
1454S	4,910,943	84	223,309	2,803,590
1455S	4,681,603	81	411,012	2,605,576

Supplementary Table 1: Assembly metrics of sequenced isolates

	Genome size (bp)	Depth (X)	N50	N#Reads (PE)
1456S	4,761,667	79	550,002	2,771,860
1457S	4,598,468	84	289,125	2,182,656
1458S	4,742,541	64	310,336	1,938,542
1459S	5,000,371	86	270,584	2,695,680
1460S	4,983,170	65	271,671	2,056,098
1461S	4,812,310	39	700,723	1,108,614
1462S	5,037,179	81	277,466	3,047,408
1463S	4,668,982	80	490,275	2,409,672
1464S	5,071,880	38	270,591	1,011,236
1465S	4,856,460	87	531,837	2,370,584
1466S	4,852,431	56	272,039	1,499,204
1468S	4,784,923	57	409,722	1,518,094
1469S	4,714,180	84	496,975	2,699,898
1470S	5,020,511	51	189,639	1,386,736
1471S	4,598,680	63	386,688	1,602,172
1472S	4,753,192	40	543,233	1,153,960
1473S	5,017,865	10	62,237	1,146,218
1474S	4,889,272	11	99,558	1,227,782
1475S	4,566,121	9	134,911	1,382,356
1476S	4,964,206	14	117,115	1,022,842
1477S	4,969,344	25	191,351	1,695,700
1478S	4,714,910	26	266,801	1,856,160
1479S	4,995,335	7	159,391	853,614
1480S	4,609,718	12	157,279	1,166,664
1481S	5,000,538	54	183,197	1,399,500
1482S	4,932,627	63	440,963	1,818,870
1483S	4,956,741	37	142,066	1,155,996

Supplementary Table 1: Assembly metrics of sequenced isolates

	Genome size (bp)	Depth (X)	N50	N#Reads (PE)
1484S	4,926,192	85	160,955	2,888,872
1485S	4,602,810	78	92,417	4,006,692
1486S	4,609,876	7	55,724	1,029,878
1487S	4,566,999	11	96,375	1,352,280
1488S	4,701,390	2	65,953	837,630
1489S	4,797,847	46	283,477	1,263,530
1490S	4,950,302	80	272,039	2,319,770
1491S	4,941,883	46	270,591	1,182,736
1492S	5,034,780	52	166,298	1,765,512
1493S	4,598,487	79	408,110	2,145,820
1494S	4,954,391	92	251,293	2,487,584
1495S	4,984,044	74	270,591	1,985,938
1496S	4,547,967	53	654,408	1,431,024
1500S	4,954,354	60	260,522	1,794,712
1501S	4,854,762	62	151,499	1,791,190
1503S	4,936,162	69	270,584	2,407,356
1504S	4,801,640	43	414,294	1,283,940
1505S	4,838,606	58	161,318	1,700,164
1506S	4,723,806	59	355,392	1,613,764
1507S	4,741,092	56	414,864	1,490,354
1508S	4,590,466	50	88,953	1,246,822
1509S	5,089,361	26	463,546	767,824
1510S	4,913,606	54	325,079	1,514,338
1511S	4,771,090	119	118,190	34,841,868
1512S	4,992,678	36	263,447	1,144,720
1513S	4,621,446	130	306,236	40,661,304
1514S	4,734,244	133	401,736	37,314,580

Supplementary Table 1: Assembly metrics of sequenced isolates

	Genome size (bp)	Depth (X)	N50	N#Reads (PE)
1515S	4,534,755	97	121,521	21,883,600
1516S	4,905,785	187	282,701	66,866,638
1517S	4,816,573	157	420,239	49,669,692
1518S	5,023,897	149	225,732	54,566,684
1519S	4,736,089	128	543,106	39,277,252
1520S	4,824,256	129	420,255	39,253,998
1521S	4,798,210	144	464,357	49,751,236
1522S	4,624,883	161	349,071	46,033,820
1523S	4,684,056	183	177,993	67,072,512
1524S	4,624,538	185	349,071	66,382,620
1525S	5,036,929	107	271,054	26,139,816
1526S	4,945,711	107	271,077	28,441,056

Supplementary Table 2: Frequency of serovars per specimen

	Abscess	Biopsy	Blood	Faeces	Synovial fluid	Urine
Monophasic Typhimurium	1	0	6	88	0	5
Enteritidis	0	0	4	24	1	2
Not characterized	0	1	6	21	0	6
Napoli	0	0	2	25	0	0
Typhimurium	0	0	1	11	0	4
Derby	1	0	0	11	0	1
London	1	0	1	9	0	1
Brandenburg	0	0	0	5	0	0
Agona	0	0	0	6	0	0
Braenderup	0	0	0	1	0	0
Chincol	0	0	0	1	0	0
Choleraesuis	0	0	2	0	0	0
Colindale	0	0	0	1	0	0
Give	0	0	0	1	0	0
Goldcoast	0	0	1	1	0	0
Hadar	0	0	0	0	0	1
Infantis	0	0	0	4	0	1
Kenya	0	0	0	1	0	0
Kimuenza	0	0	0	1	0	0
Mbandaka	0	0	0	2	0	0
Meleagridis	0	0	0	1	0	0
Montevideo	0	0	1	1	0	0
Muenchen	0	0	0	0	0	1
Newport	0	0	0	1	0	0
Panama	0	0	1	1	0	0
Rissen	0	0	0	2	0	0
Saintpaul	0	0	0	1	0	0

Supplementary Table 2: Frequency of serovars per specimen

	Abscess	Biopsy	Blood	Faeces	Synovial fluid	Urine
Sandiego	0	0	0	1	0	0
Schwarzengrund	0	0	0	1	0	0
Stanley	0	0	0	2	0	0
Stratchona	0	0	0	2	0	0
Thompson	0	0	0	0	0	1
Typhi	0	0	2	1	0	0

Supplementary Table 3: Frequency of serovars per year

	2021	2022	2023	2024
Monophasic Typhimurium	26	22	32	20
Enteritidis	15	6	6	4
Typhimurium	9	2	3	2
Brandenburg	0	3	0	2
Not characterized	6	7	11	10
London	4	1	2	5
Kimuenza	0	1	0	0
Infantis	0	1	1	3
Agona	2	2	2	0
Napoli	8	3	10	6
Mbandaka	0	2	0	0
Choleraesuis	0	2	0	0
Rissen	1	1	0	0
Goldcoast	0	1	1	0
Derby	2	0	5	6
Newport	0	0	1	0
Montevideo	0	1	1	0
Kenya	0	0	0	1
Give	0	0	0	1
Saintpaul	0	0	0	1
Panama	0	1	0	1
Hadar	0	1	0	0
Thompson	0	0	1	0
Braenderup	0	0	0	1
Chincol	1	0	0	0
Meleagridis	1	0	0	0
Stratchona	1	0	1	0

Supplementary Table 3: Frequency of serovars per year

	2021	2022	2023	2024
Typhi	1	0	1	1
Sandiego	0	1	0	0
Schwarzengrund	0	1	0	0
Stanley	0	0	1	1
Colindale	0	0	0	1
Muenchen	0	0	1	0
